# Individual and community-level determinants of overweight and obesity among urban men: Further analysis of the Ethiopian demographic and health survey

**DOI:** 10.1371/journal.pone.0259412

**Published:** 2021-11-04

**Authors:** Yohannes Tekalegn, Zinash Teferu Engida, Biniyam Sahiledengle, Heather L. Rogers, Kenbon Seyoum, Demelash Woldeyohannes, Birhan Legese, Tadesse Awoke Ayele

**Affiliations:** 1 Department of Public Health, School of Health Science, Goba Referral Hospital, Madda Walabu University, Goba, Ethiopia; 2 Biocruces Bizkaia Health Research Institute, Barakaldo, Spain and IKERBASQUE Basque Foundation for Science, Bilbao, Spain; 3 Department of Midwifery, School of Health Science, Goba Referral Hospital, Madda Walabu University, Goba, Ethiopia; 4 Department of Public Health, School of Medicine and Health Science, Wachemo University, Hosana, Ethiopia; 5 Department of Public Health, School of Medicine and Health Science, Ambo University, Ambo, Ethiopia; 6 Department of Epidemiology and Biostatistics, University of Gondar, Gondar, Ethiopia; University of Zurich, SWITZERLAND

## Abstract

**Background:**

Overweight and obesity have become a serious public health problem in both developed and developing countries, particularly in urban areas. However, there are limited studies conducted to identify the risk factors of overweight and obesity in Ethiopia, especially among men. Therefore, this study aimed to assess individual and community level determinants of overweight and obesity among urban men in Ethiopia.

**Methods:**

This study used the 2016 Ethiopian Demographic and Health Survey (EDHS) data. A weighted sample of 2259 urban men aged 15–59 years were included in this analysis. A multilevel logistic regression model was used to assess the determinants of overweight and obesity among the study participants.

**Results:**

Men aged 30–44 years old (AOR = 3.1, 95% CI: 2.3–4.11), 45–59 years old (AOR = 4.8, 95% CI: 3.4–6.9), married (AOR = 1.7, 95% CI: 1.3–2.2), with secondary education (AOR = 2.7, 95% CI: 1.6–4.7), with higher education (AOR = 3.6, 95% CI: 2.1–6.2), watching television at least once a week (AOR = 1.7, 95% CI: 1.1–2.7), being from high rich communities (AOR = 2.4, 95% CI: 1.5–3.7), and living in three metropolises (Addis Ababa, Harari, Diredawa) were more likely to be overweight or obese (AOR = 1.8, 95% CI: 1.1–2.9). However, currently unemployed men were less likely to be overweight or obese (AOR = 0.5, 95% CI: 0.3–0.7).

**Conclusion:**

Being older age, being married, having higher educational status, having higher frequency of watching television, being residents of three metropolises (Addis Ababa, Harari, and Diredawa), and being from high rich communities were found to be predictors of overweight and obesity in Ethiopian men. Therefore, it is essential to design strategies and programs to reduce or prevent overweight and obesity with special focus on the identified risk factors.

## Introduction

The World Health Organization (WHO) has defined overweight and obesity as abnormal or excessive accumulation of fat in the body that negatively affect human health [1]. The WHO distinguishes between overweight and obesity using a crude population measure based on body mass index (BMI; a person’s weight in kilograms divided by the square of his/her height in meters). The classification of obese is generally made when a person’s BMI is ≥ 30, whereas overweight is ≥ 25 but < 30 [2].

The prevalence of overweight and obesity is rising globally in both developing and developed countries, particularly among those with rapid socio-cultural changes [3–8].Worldwide overweight and obesity was estimated to affect more than 1.9 billion adults in 2016 [9]. Higher BMI is a major risk factor of non-communicable diseases (NCDs) such as type 2 diabetes, cardiovascular diseases and some cancers [10–15]. In 2015, overweight and obesity contributed to about 4 million deaths and 40 million Disability-Adjusted Life-Years (DALYs) for adults worldwide [16].

Many low and middle-income countries are facing additional burden from NCDs while dealing with the challenges of infectious diseases like malaria, tuberculosis and HIV/AIDS [9, 17]. In Ethiopia, proportionate death from NCDs has recently increased, causing about 42% of total deaths. Among these, 27% are premature deaths occurring before 70 years of age. DALYs due to NCDs in the country was 69% in 2015, which is more than double that of communicable disease and maternal, neonatal and nutritional problems together [18, 19].

Prior to 2000, overweight and obesity did not receive much public health attention in Ethiopia. But recently, the prevalence of adult overweight and obesity has increased [20]. According to Ethiopian Demographic and Health Survey (EDHS) reports, the prevalence of overweight and obesity among adult men increased from 2.5% in 2011 to 3.5% in 2016. However, there is high urban-rural disparity in the trend of overweight and obesity. The prevalence in urban areas increased from 7.6% in 2011 to 12.4% in 2016, while the rural areas maintained a prevalence of less than 1% during this time span [21, 22]. A recent systematic review and meta-analysis conducted in Ethiopia reported that the prevalence of overweight and obesity in urban areas was 22.4% and 6.2%, respectively [23].

In Ethiopia, studies have reported the prevalence and determinants of overweight and obesity among reproductive age women [24–26] and adults in general. However, most prior research has been conducted with restricted samples from specific districts. Without nationally representative samples, the generalizability of the results is questionable. Also, most studies have focused on individual level determinants of overweight/obesity [27–29]. Yet, overweight and obesity have a multitude of determinants resulting from genetic, behavioral, cultural, and environmental factors [30–32] that must be accounted for, which makes prevention difficult to address. In addition, metabolic risk factors of overweight and obesity differ among men and women [33, 34].

The present study analyzes nationally representative EDHS data, employing multilevel analysis to assess the individual and community level determinants of overweight and obesity among adult men residing in urban Ethiopia. Identifying determinants at both levels will assist in the prevention and control efforts of these emerging public health challenges in Ethiopia. The information can also be used as a baseline evidence for public health program planners, policymakers, researchers and organizations who are working on prevention of chronic non-communicable diseases. Results can assist community members by providing information on the risk factors of overweight and obesity among urban men.

## Methods and materials

### Data sources

This cross-sectional study was conducted using the 2016 Ethiopian Demographic and Health Survey (EDHS) dataset, a nationally representative survey. It was implemented by the Central Statistical Agency (CSA). Data collection took place from January 18, 2016, to June 27, 2016 [22]. The study is conducted in compliance to the STROBE cross sectional reporting guidelines [35]

### Population and sampling procedures

The 2016 EDHS data was collected using a sampling frame from the Ethiopian population and housing census which was conducted in 2007 by the Ethiopian CSA. The survey was designed to represent the country as a whole, for urban and rural areas separately. All of the nine administrative regions and the two city administrations in Ethiopia were included in the survey. A two-stage stratified cluster sampling was used. Each region and one city administration were stratified into urban and rural, except Addis Ababa, which is entirely urban. In a total, 21 sampling strata were created. A total of 645 enumeration areas (202 in urban areas and 443 in rural areas) were selected with probability proportional to size of enumeration areas and with independent selection in each sampling stratum. In the second stage, a fixed number of 28 households per cluster were selected with an equal probability systematic selection from newly created household listing [22].

### Data collection and anthropometric measurements

EDHS survey data was collected using structured interviewer administered questionnaire. All men aged 15–59 years who were either permanent residents of selected households or visitors who stayed in the household the night before the survey were eligible to be interviewed for the male version of the questionnaire. The interview was carried out using tablet computers to record responses. Height and weight of all men aged 15–59 were measured. Weight was measured using light weight SECA mother-infant scales with digital screen designed and manufactured under guidance of UNICEF. Height measurements were carried out using a Shorr measuring board. A detailed explanation of data collection procedures can be found elsewhere [22].

### Outcome variable

Overweight and obesity is the outcome variable of this study, which was derived from body mass index (BMI) data. BMI was calculated as weight in kilograms divided by height in meters squared. According to the World Health Organization classification, BMI of <18.5 kg/m^2^, 18.5–24.99 kg/m^2^, 25–29.99 kg/m^2^, and ≥30 kg/m^2^ was categorized as underweight, normal, overweight, and obese respectively [2]. Individuals with either overweight or obesity were combined into one category and coded as “1” and others were coded as “0”.

### Independent variables

The independent variables were classified as either individual level or community level factors. Individual level variables included variables such as age (categorized into 15–29 years, 30–44 years, 45–59 years), marital status (categorized as single, married/living with partner, and widowed/divorced/separated), educational level (labeled as no education, primary education, secondary education, and higher than secondary education), current employment status (categorized as employed or unemployed), frequency of watching television (categorized as not at all, less than once a week, at least once a week), listening to the radio (categorized as not at all, less than once a week, at least once a week), and reading magazines (categorized as not at all, less than once a week, at least once a week).

Community level variables were administrative region and level of “rich” households in the community level. Ethiopia was divided into nine regions and two city administrations. These regions were re-categorized based on the settings associated with the prevalence of overweight and obesity [36]. Accordingly, regions were defined as: “Metropolis” (containing Addis Ababa, Harari and Diredawa), Tigray, Amhara, Oromia, South nation, nationalities and people’s region (SNNPR), and “Other” (Afar, Benshangul-Gumuz, Gambela and Somali). Level of “rich” households in the community was defined as the proportion of men in richer and richest households within each cluster. The household wealth index variable was used, which indicates a household’s cumulative living standard and includes items like owning a bicycle, television, or refrigerator. In the EDHS data, the majority of urban dwellers are very rich. This variable was categorized as high rich or low rich using a median split. Then the proportion of high rich households for each cluster was calculated.

### Data analysis procedures

Data analysis was conducted using STATA version 14. To calculate the overweight or obesity status of men, we merged anthropometric variables from household members recode (PR file) to the men’s recode (MR file) using the cluster, household and line numbers [37]. After merging both datasets, a weighted sample of 2259 men aged 15–59 years living in urban areas were included in the final analysis. Sampling weight was used during data analysis to adjust for non-proportional allocation of sample and possible differences in response rates across regions included in the survey. No missing data was present. Due to the hierarchical nature of the EDHS data and presence of intra-class correlation (ICC) multilevel logistic regression was used instead of ordinary logistic regression. Both bi-variable and multi-variable multilevel logistic regression was performed to assess the independent effect of the individual and community level variables on overweight and obesity. Independent variables with p-values less than 0.05 in bi-variable analysis were entered into the multi-variable logistic regression. Fixed effects were reported using adjusted odds ratio (AOR) with 95% confidence intervals (CI). Random effect parameters were measured by ICC, proportional change in variance (PCV) and median odds ratio (MOR), which measure the variability between clusters in the multilevel models. ICC explains the cluster variability, while MOR can quantify unexplained cluster variability (heterogeneity). MOR translates cluster variance into OR scale. In the multilevel model, PCV can measure the total variation due to factors at the community and individual level [38]. Model comparison was conducted for the null model (model without explanatory variables), model 1 (Model adjusted for individual level factors), model 2 (Model adjusted for community level factors), and model 3 (Final model adjusted for both individual and community level factors). Multi-collinearity among independent variables was checked using variance inflation factors (VIF).

### Ethical considerations

The Ethiopian Demographic and Health Survey was conducted after the approval of the Ethiopian National Research Ethics Review Committee. Permission to use the 2016 EDHS database for further analysis was sought from http://www.dhsprogram.com and no ethics committee approval was necessary. The data was analyzed and reported in aggregate; household and individual identifiers were not reported in the dataset.

## Results

### Characteristics of the study participants

A weighted sample of 2259 urban men aged 15–59 were included in the analysis. One thousand two hundred two (53.2%) study participants were in age range of 15–29 years old. One thousand one hundred four (48.8%) were single. Most of the study participants, 2066 (91.5%) received primary education or above. In terms of residence region, 592 (26.2%), 520 (23.0%), and 607 (26.9%) were from Oromia, Amhara, the three metropolises, respectively. Out of all study participants, 2118 (93.7%) were either in richer or richest quintiles (Table 1).

**Table 1:** Sociodemographic characteristics of men aged 15–59 years in urban Ethiopia, 2016 EDHS, (N^a^ = 2259).

Variables	Frequency	Percent
**Age in years**		
15–29	1202	53.2
30–44	737	32.6
45–59	320	14.2
**Marital status**		
Single	1104	48.8
Married/living with partner	1091	48.3
Divorced/widowed/separated	64	2.7
**Religion**		
Orthodox	1338	59.2
Muslim	450	19.9
Protestant	453	20.0
Catholic	4	0.2
Others	14	0.6
**Educational level**		
No education	193	8.5
Primary	639	28.3
Secondary	686	30.4
Higher than secondary	741	32.8
**Current work status**		
Employed	1827	80.9
Unemployed	432	19.1
**Wealth index**		
Poorest	71	3.1
Poorer	35	1.6
Middle	35	1.6
Richer	64	2.8
Richest	2054	90.9
**Frequency of watching television**		
Not at all	358	15.8
Less than once a week	436	19.3
At least once a week	1466	64.9
**Frequency of listening to the radio**		
Not at all	622	27.5
Less than once a week	495	21.9
At least once a week	1142	50.6
**Frequency of reading newspapers or magazines**		
Not at all	1065	47.1
Less than once a week	672	29.8
At least once a week	522	23.1
**BMI Classification**		
Under weight	552	24.5
Normal	1397	61.8
Over weight	267	11.8
Obese	43	1.9
**Region of residence**		
Tigray	156	6.9
Amhara	520	23.0
Oromia	592	26.2
SNNPR^b^	260	11.5
The three metropolises^c^	607	26.9
Others^d^	124	5.5
**Community rich level**		
Low rich	550	24.3
High rich	1710	75.7

Notes:

^a^ Total weighted sample;

^b^ South nation, nationalities and peoples region;

^c^Addis Ababa, Diredawa and Harari;

^d^Afar, Somali, Benishangul-Gumuz and Gambela.

### Multilevel models comparison

The community-level variance (ICC) was 11.3%, indicating there was a considerable difference of prevalence of overweight and obesity at the community-level; the difference declined to 2.3% after individual and community level variables were controlled in the combined model. Median odds ratio (MOR) for overweight and obesity was 1.8 in the null model which indicates that there was variation between clusters. If individuals are randomly selected from two different clusters, individuals at the cluster with a higher risk of overweight or obesity had 1.8 times higher risk of being overweight or obese as compared with individuals at cluster with a lower risk of overweight or obesity. The models were compared using Log-likelihood and models 3 (Final model adjusted for both individual and community level factors) was selected, having highest log-likelihood (-1203.42) (Table 2).

**Table 2:** Model comparators and random effect parameters of the study to assess the determinants of overweight and obesity among men aged 15–59 years in urban Ethiopia, 2016 EDHS.

	Null model	Model 1	Model 2	Model 3
ICC (%)	11.29	5.72	4.71	2.27
MOR	1.84 (1.83–1.86)	1.52 (1.50–1.56)	1.46 (1.44–1.49)	1.29 (1.23–1.36)
PCV	Reference	52.3	61.2	81.8
Log-likelihood	-1435.20	-1227.06	-1398.93	-1203.42
AIC	2874.39	2488.11	2823.87	2462.85

**Notes**: ICC: Intra cluster correlation; MOR: Median odds ratio; PCV: Proportional change in variance; AIC: Akaike information criteria; Null model: Baseline model without explanatory variables; Model 1: Model adjusted for individual level factors; Model 2: Model adjusted for community level factors; Model 3: Final model adjusted for both individual and community level factors.

### Determinants of overweight and obesity

In the final multi-variable multilevel logistic regression model: age, marital status, educational level, employment status, watching television, region of residence, and community rich level were significantly associated with being overweight or obese.

The odds of being overweight or obese was higher among men aged 30–44 years (AOR = 3.1, 95% CI: 2.3–4.1) and 45–59 years (AOR = 4.8, 95% CI: 3.4–6.9) compared to the men aged 15–29 years. With regard to marital status, likelihood of being overweight or obese was higher among married men or those living with partner (AOR = 1.7, 95% CI: 1.3–2.2) compared to singles. The odds of being overweight or obese was higher among men with secondary education (AOR = 2.7, 95% CI: 1.6–4.7) and higher education (AOR = 3.6, 95% CI: 2.1–6.2) compared to men with no education. Unemployed men were less likely to be overweight or obese (AOR = 0.5, 95% CI: 0.3–0.7) compared to employed men. The likelihood of being overweight or obese was higher among men watching television at least once a week (AOR = 1.7, 95% CI: 1.1–2.7) compared to men not watching television.

Among community level variables, men from high rich community were more likely to be overweight or obese (AOR = 2.4, 95% CI: 1.5–3.7) compared to low rich community. In terms of place residence by administrative regions, men living in the three metropolises (Addis Ababa, Harari and Diredawa) were more likely to be overweight or obese (AOR = 1.8, 95% CI: 1.1–2.9) compared to those living in the Tigray region (Table 3).

**Table 3:** Bi-variable and multi-variable multilevel logistic regression analysis to assess the determinants of overweight and obesity among men aged 15–59 years in urban Ethiopia, 2016 EDHS.

Variables	Overweight/obesity		COR (95% CI)	AOR (95% CI)
	Yes (%)	No (%)		
	(n = 310)	(n = 1949)		
**Age in years**				
15–29	71 (5.9)	1132 (94.1)	1	1
30–44	146 (19.9)	590 (80.1)	4.4 (3.5–5.6) *	3.1 (2.3–4.11) **
45–59	93 (28.9)	227 (71.1)	6.7 (5.1–8.9) *	4.8 (3.4–6.9) **
**Marital status**				
Single	72 (6.5)	1032 (93.5)	1	1
Married/living with partner	225 (20.6)	866 (79.4)	3.9 (3.1–5.6) *	1.7 (1.3–2.2) **
Widowed/divorced/separated	13 (19.9)	51 (80.1)	2.8 (1.7–4.6) *	1.1 (0.6–1.9)
**Education level**				
No education	17 (8.9)	176 (91.1)	1	1
Primary	50 (7.9)	589 (92.1)	1.2 (0.7–2.2)	1.4 (0.8–2.4)
Secondary	94 (13.8)	592 (86.2)	2.1 (1.2–3.5) *	2.7 (1.6–4.7) **
Higher	148 (19.9)	593 (80.1)	3.2 (1.9–5.3) *	3.6 (2.1–6.2) **
**Current employment status**				
Employed	288 (15.8)	1539 (84.2)	1	1
Not employed	22 (5.1)	410 (94.9)	0.2 (0.1–0.3) *	0.5 (0.3–0.7) **
**Frequency of watching television**				
Not at all	20 (5.6)	338 (94.4)	1	1
Less than once a week	40 (9.1)	396 (90.9)	1.6 (0.9–2.5)	1.1 (0.6–1.8)
At least once a week	250 (17.1)	1216 (82.9)	2.9 (1.9–4.4) *	1.7 (1.1–2.7) **
**Frequency of listening to the radio**				
Not at all	73 (11.7)	549 (88.3)	1	1
Less than once a week	58 (11.8)	436 (88.2)	1.3 (0.9–1.8)	0.9 (0.7–1.3)
At least once a week	179 (15.7)	964 (84.3)	1.7 (1.3–2.2) *	0.8 (0.6–1.1)
**Frequency of reading newspapers or magazines**				
Not at all	106 (9.9)	959 (90.1)	1	1
Less than once a week	105 (15.6)	567 (84.4)	1.6 (1.3–2.1) *	1.1 (0.9–1.5)
At least once a week	99 (18.9)	423 (81.1)	2.4 (1.9–3.1) *	1.2 (0.9–1.6)
**Community rich level**				
Low rich	24 (4.4)	525 (95.6)	1	1
High rich	286 (16.7)	1424 (83.3)	4.1 (2.7–6.0) *	2.4 (1.5–3.7) **
**Region of residence**				
Tigray	18 (11.7)	137 (88.3)	1	1
Amhara	40 (7.7)	479 (92.3)	0.6 (0.3–1.4)	0.8 (0.4–1.6)
Oromia	90(15.2)	502 (84.8)	1.5 (0.7–3.2)	1.5 (0.7–2.9)
SNNPR^b^	26(9.9)	234(90.1)	1.2 (0.5–2.6)	1.6 (0.7–3.4)
Three metropolis^c^	124 (20.4)	483(79.6)	1.8 (1.1–3.1) *	1.8 (1.1–2.9) **
Others^d^	12 (9.3)	112 (90.7)	0.8 (0.4–1.4)	1.4 (0.8–2.4)

Notes:

* significant at P<0.05 (crude);

**significant at P<0.05 (adjusted);

^b^South nation, nationalities and peoples region;

^c^Addis Ababa, Diredawa and Harari;

^d^Afar, Somali, Benishangul-Gumuz and Gambela.

## Discussion

This study used multi-level mixed effect logistic regression model to assess the individual and community level determinants of overweight and obesity among urban men aged 15–59 years old in Ethiopia. Individual level factors such as age, marital status, educational level, current employment status, and frequency of watching television were significantly associated with overweight and obesity. Among the community-level factors examined, region of residence and community rich level were significantly associated with overweight and obesity.

The likelihood of overweight and obesity was significantly higher among men aged 30–44 years and 45–59 years old, compared to those 15–29 years old. In this study the risk of overweight and obesity increases as age increases. Similar study in United States reported that weight and body mass index rises with age [39]. The positive association between age and overweight/obesity is supported by several studies in Ethiopia, as well as one in Iran [27, 28, 40]. Likewise, men with secondary and higher education were more likely to be overweight or obese compared to men with no education. The likely justification for this finding is that educated people in developing countries tend to have better socio-economic status and are more likely to follow a western lifestyle, which includes consumption of energy dense food, less physical activity, and more sedentary activities, which may lead to increased risk of being overweight or obese [41]. A systematic review conducted by Cohen et.al, reported that educational attainment is positively associated with obesity in developing countries and inversely associated in developed countries [42]. Interestingly, the likelihood of overweight or obesity was lower among unemployed men compared to employed men, suggesting that employed men may be more sedentary, although further research is warranted to test this hypothesis.

The odds of overweight or obesity were higher among married men compared to their unmarried counterparts. Married men were more likely to be in the older age group, but this relationship existed after controlling for age. Research in Iranian adults suggests that married men are less likely to be concerned about their body shape, more likely to change their dietary pattern, and might have some other environmental exposure related to weight [40]. Another study conducted in the United States suggests that married men are more likely to be obese than never married and previously married men. The study explained the association by suggesting “married men are more likely to eat regularly or abundantly when they have a wife and therefore be fatter when they are married” [43]. This finding is supported by evidence from Ethiopia and elsewhere [40, 44, 45].

Men watching television at least once a week were at higher odds of being overweight or obese compared to their counterparts who did not watch television at all. Men who have a habit of watching television may be more likely to be sedentary, and could be more exposed to advertisements for unhealthy foods, alcohol and beverages, which may lead to increases in the risk of overweight and obesity. Further research is needed to test this hypothesis. The finding on the relationship between television watching and overweight or obesity is supported by previous studies in many countries, including Nepal [46], Bangladesh [47], Myanmar [48], Australia [49], the USA [50], Iran [51] and China [52].

Among the community-level factors assessed for potential association with overweight and obesity, community-level richness and region of residence were significantly associated with overweight and obesity. Men from a high rich community were 2.4 times more likely to be overweight or obese compared to those from a low rich community. This might be partly explained by lifestyle and other socioeconomic factors. It is possible that wealthier men in urban settings are more likely to consume high energy dense food, eat large portions of food because they can afford to do so, and may be less likely to engage in physically demanding work. Furthermore, urban men with higher socioeconomic status own and use cars, so that they walk less often [53]. The positive association between wealth index and overweight/obesity is supported by previous studies in Ethiopian women and adults from Ethiopia, India, and Bangladesh [24, 26, 27, 29, 36, 54–56].

Men residing in the three metropolises (Addis Ababa, Harari, and Diredawa) had higher odds of being overweight or obese compared to those residing in Tigray. Men living in metropolises may be more likely to consume western style foods and follow sedentary lifestyles, which increases the likelihood of overweight and obesity. This finding is supported by several previous studies in Ethiopia and elsewhere [24, 28, 36, 40, 57, 58].

## Strength and limitations of the study

- This is the first study to assess the determinants of overweight and obesity among urban men in Ethiopia using Demographic and Health Survey data which is a nationally representative data.
- The study used multilevel logistic regression model which enhances the accuracy of estimates.
- Risk factors for overweight vs. obesity are not examined separately in this analysis.
- Due to secondary nature of the data, some important variables which can affect the likelihood overweight and obesity such as dietary behavior and exercise were not measured. Furthermore, indicators of central obesity like waist and hip circumferences were not measured by this survey.
- Due to cross-sectional nature of the data, cause-effect relationship between outcome and independent variables cannot be established.

## Conclusion

This study assessed individual and community level sociodemographic correlates of overweight and obesity among urban men in Ethiopia. Individual level factors like being older age, married, higher educational status, and increased frequency of watching television were significantly associated with overweight and obesity. Policy makers and concerned public health authorities should design strategies and programs on behavioral, dietary and lifestyle interventions which can help in reducing the risk of overweight/obesity directed at these at-risk groups of the population. Community level factors such as being residents of three metropolises (Addis Ababa, Harari, and Diredawa) and being from high rich communities were associated with higher odds of overweight and obesity. Men residing in these cities are should be targeted for mass sports and lifestyle modification programs designed to reduce overweight/obesity and/or maintain normal body weight. Obesity prevention programs and strategies in Ethiopia should be developed with these risk factors and at-risk groups in mind.
